# Flavonoids Modulate *Aspergillus flavus* Proliferation and Aflatoxin Production

**DOI:** 10.3390/jof8111211

**Published:** 2022-11-16

**Authors:** Lina Castano-Duque, Matthew D. Lebar, Carol Carter-Wientjes, David Ambrogio, Kanniah Rajasekaran

**Affiliations:** United States Department of Agriculture—Agriculture Research Services, New Orleans, LA 70124, USA

**Keywords:** *Aspergillus*, aflatoxin, quercetin, luteolin, apigenin

## Abstract

Aflatoxins are carcinogenic mycotoxins produced by *Aspergillus flavus*. They contaminate major food crops, particularly corn, and pose a worldwide health concern. Flavonoid production has been correlated to resistance to aflatoxin accumulation in corn. The effects of flavonoids on fungal proliferation and aflatoxin production are not well understood. In this study, we performed bioassays, fluorescence and scanning electron microscopy, and total antioxidant analysis to determine the effects of three flavonoids (apigenin, luteolin, and quercetin) on proliferation and aflatoxin production in *A. flavus* NRRL 3357. Results showed that concentrations of apigenin and luteolin modulated fungal proliferation and aflatoxin production in a dose-dependent manner, leading to inhibition or promotion of proliferation and toxin production. Microscopy studies of fungi exposed to flavonoids showed mycelial cell wall disruption, abnormal cell wall invaginations, and tears. Fluorescent enhancement of apigenin and luteolin using Naturstoff reagent A showed that these chemicals localized in sphere-like structures on the mycelia surface. Fungi exposed to low concentrations of apigenin, luteolin, and quercetin lowered the total antioxidant capacity in the environment compared to controls. Our results indicate that flavonoids disrupt cell wall integrity and may localize in vesicle-like structures. We hypothesize that flavonoids could act as potential signaling molecules at low concentrations and change the oxidative state of the microenvironment, either or both of which may lead to reduction of fungal proliferation and aflatoxin production.

## 1. Introduction

The fungus *Aspergillus flavus* colonizes fatty-acid-rich food and feed crops and contaminates them with aflatoxins, which are potent carcinogens that are highly regulated by the Food and Drug Administration (FDA) [1]. Corn, a major food and feed crop grown worldwide, is highly susceptible to *A. flavus* infection and aflatoxin contamination. Identification of plant molecules that decrease aflatoxin contamination is one tactic of a multipronged strategy to improve plant breeding programs and aid in the development of broad, integrated pest management programs. Flavonoids are phenolic-derived phytochemicals with a benzo-γ-pyrone structure and contain subclasses such as flavonols, flavones, and flavanones. High levels of phenolic compounds in corn lead to resistance against fungal growth [2] and lower levels of mycotoxin contamination in kernels [3]. Furthermore, flavonoids play a wide variety of biological roles in plants and other organisms [4,5]. We previously determined that corn lines resistant to aflatoxin contamination produce more flavonoids upon *A. flavus* infection [6]. However, the molecular mechanism that links flavonoids to regulation of fungal growth and production of aflatoxin is yet to be understood.

Flavonoids have broad biological activities, such as antimicrobial, cytotoxic, anti-inflammatory, and cancer-preventing roles [7]. Recent research on the effects of flavonoids in *Aspergillus parasiticus* treated in vitro with a micromolar flavonoid mixture (naringin, neohesperidin, and quercetin) found aflatoxin accumulation decreased by 85–100% [8]. In vitro studies showed that *Fusarium culmorum* and *Fusarium graminearum* metabolized naringenin, apigenin, luteolin, kaempferol, and quercetin to flavonoid derivatives and produced significantly less mycotoxin [9]. Interestingly, in some cases, apigenin treatment has been shown to increase mycotoxin content in the media [9]. In *Fusarium oxysporum* f. sp. *lycopersici*, antimicrobial effects have been observed with flavonoid treatments [10]. Overall, in *Fusarium* species, there is evidence that possible antioxidant metabolites can lead to a decrease in mycotoxin production [11]. There is evidence that *A. flavus* NRRL 3357 treated with anthocyanidins and flavonoids decrease aflatoxin B_1_ production [12] and growth of the fungus [13]. Corn has been shown to produce O-methylflavonoids in response to fungus infection, and these compounds showed antifungal activity to several fungal species [14]. Furthermore, flavonoids extracted from corn exhibited antimicrobial [15] and insecticidal [16] activities.

In mammals, flavonoids and their derivatives can behave as antioxidants and signaling molecules capable of altering gene expression; however, their mode of action is not well understood [17]. Flavonoid interactions with proteins, such as mitogen-activated protein kinase (MAP kinase), have been implicated in altering gene expression [17]. In plants, flavonoids are capable of scavenging ROS [18] and can act as endogenous regulators of auxin movement, thus linking them to plant developmental regulation. Intriguingly, endogenous nanomolar concentration levels of flavonoids in many species are thought to be too low to serve as effective ROS quenchers, so it is hypothesized that flavonoids serve as signaling molecules [19]. Resistant corn infected with *A. flavus* contained flavonoids in the low micromolar to nanomolar range (naringenin ~2 µM, luteolin ~0.1 µM, and luteolin-glycoside ~20 nM), while susceptible infected corn had significantly lower concentrations of each flavonoid [6]. The variation of flavonoid levels in resistant and susceptible corn lines could indicate that flavonoid type and levels may act as potential scavengers or signaling molecules. The objective of this research was to determine the effects of three flavonoids (apigenin, luteolin, and quercetin) on *A. flavus* growth and aflatoxin production. 

## 2. Materials and Methods

***Bioassays:*** Two *A. flavus* strains were used: NRRL 3357 as described before [20], herein called AF3357, and *A. flavus 70* transformed to express green fluorescent protein (GFP) [21], herein called AF70 (used for fluorescence microscopy). Flavonoids (apigenin, luteolin, and quercetin) were purchased from Indofine Chemical Co. (Hillsborough Township, NJ, USA). To a 24-well plate (Costar), we added 1 mL of fungal inoculum (1 × 10^4^ CFU/mL in 1% potato dextrose broth (PDB, DIFCO) with 0.0001% Triton-X pH 6.0) [22,23] and each of the flavonoids at various concentrations of weight to final volume in inoculum (0, 0.1, 0.01, 0.001, 0.0001, and 0.00001 µg/µL, see Supplementary Materials for µM concentrations) with 1% dimethyl sulfoxide (DMSO, J.T. Baker). The flavonoid stock solutions were prepared in 100% DMSO prior to adding them to 1 mL of fungal inoculum. These compounds were soluble in 100% DMSO and remained soluble after adding them to the fungal inoculum. However, after 24 h incubation at 31 °C, we observed formation of crystals at the bottom of the wells in the highest concentrations (0.1 µg/µL). Thus, we decided not to extend the experiment beyond 24 h. Both flavonoid treatments and controls were exposed to the same concentration of DMSO (140 mM). Three replicate wells per flavonoid were used, with the control containing 1% DMSO, for a total of 18 wells per isolate. Well plates were incubated at 31 °C in the dark without shaking. After 12 h of incubation, 50 µL aliquots from each well were plated on potato dextrose agar (PDA, DIFCO) and incubated in the dark at 31 °C for 18 h. After 18 h in PDA plates, viable colonies were counted to determine proliferation as a measurement of fungal cells recovery response to flavonoid exposure as previously described [13] (also see Supplementary Materials). The PDA plates were incubated for a total of 68 h (~3 d) in the dark at 31 °C, then photographed and stored at −20 °C for further mycotoxin analysis. After 24 h of incubation, the remaining cultures in the 24-well plates with PDB and flavonoids were fixed by adding glutaraldehyde to a final 4% concentration in the media and prepared for microscopy analysis. 

***Aflatoxin determination and UPLC:*** All chemicals and solvents were purchased from Sigma-Aldrich (St. Louis, MO, USA) unless otherwise noted. Three agar plugs (5 mm diameter and ~3 mm depth) were excised from the center of five colonies, which were selected at random, of each treatment grown on PDA using a spectrum transfer tube (Thermo Fisher Scientific, Waltham, MA, USA). The plugs were treated with 1 mL of (10:20:30 methanol/dichloromethane/ethyl acetate) on an orbital shaker overnight at room temperature. The extracts were filtered through cotton plugs, and the filtrates were concentrated using a Savant speedvac (Thermo Scientific). Each extract was redissolved in methanol (1 mL), particulates were removed via centrifuge, and the supernatant was analyzed using a Waters (Milford, MA, USA) ACQUITY UPLC system (40% methanol in water, BEH C18 1.7 μm, 2.1 mm × 50 mm column) using fluorescence detection (excitation: λ = 365 nm, emission: λ = 440 nm). Samples were diluted to 10-fold if the aflatoxin signal saturated the detector. An analytical standard (Sigma-Aldrich) was used to identify and quantify aflatoxin B_1_ (AFB_1_). Aflatoxin content was expressed in ng AFB_1_/g agar (ppb). 

***Microscopy:*** Fluorescence and bright field images of both AF70 and AF3357 were taken using an SMZ25 stereoscope (Nikon Metrology Inc., Brighton, MI, USA) and an upright Eclipse Ni-E microscope (Nikon Metrology Inc.) as previously described by Rajasekaran et al. (2008). Scanning electron microscope (SEM) observations were made on a HITACHI S-4800 FEG CRYO-SEM (Hitachi High-Tech America, Inc., Los Angeles, CA, USA). For SEM analysis, AF3357 was incubated with quercetin, apigenin, and luteolin (0.01 and 0.00001 µg/µL) for 24 h at 31 °C in the dark (1 × 10^4^ CFU/mL initial concentration into 1% PDB medium pH 6.0 with 0.0001% Triton-X). After incubation, the samples were fixed with 4% glutaraldehyde. Fixed samples were dehydrated by passage through increasing concentrations of ethanol solutions (25–100%), then critical point dried and coated with graphite (usually 100 or 200 Å). 

***MTT assay for REDOX potential:*** To assess cell viability as a function of redox potential, we used a modified MTT (3-(4,5-dimethylthiazol-2-yl)-2,5-diphenyltetra-azolium bromide assay) assay [13]. Spores of AF3357 or AF70 were inoculated at 1 × 10^4^ CFU/mL into 1% PDB medium pH 6.0 with 0.0001% Triton-X. Next, 200 µL of spore mix was added to each well in 96-well plates, and flavonoids in 1% DMSO final concentration were added to each well. Four replicates per treatment were used as well as absorbance blanks (no fungus) and control (1% DMSO). After incubating for 24 h at 31 °C in the dark without shaking, absorbance was measured at 600 nm to determine growth with a Biotek Synergy Neo2 multimode reader (Agilent, Santa Clara, CA, USA). Then, 10 µL of MTT (Invitrogen M64941-1G, 2.5 mg/mL) was added to each well. The plates were allowed to incubate at 31 °C for 3.5 h with shaking in the dark. The reaction was terminated by the addition of DMSO (100 µL), and the plate was agitated to redissolve the formed crystals for 30 min. The absorbance of each well was assessed at 560 nm. Ratio of MTT to growth was determined as OD (560 nm)/OD (600 nm) and plotted in relation to concentration of flavonoids.

***Naturstoff reagent A (DPBA) staining for flavonoid staining:*** We used DPBA (diphenylboric acid 2-aminoethyl ester) assay as a fluorescent cross-linking analysis that specifically binds to flavonoids to determine localization of these molecules in the fungi. AF3357 was incubated for 24 h at 31 °C in the dark (1 × 10^4^ CFU/mL initial concentration into 1% PDB medium pH 6.0 with 0.0001% Triton-X). Then, 50 µL of 0.5% (*w*/*v*) DPBA (Sigma D9754-5G) in methanol was added to each well for flavonoid staining [24]. DPBA staining allows flavonoids to be visualized in vivo with fluorescence detection; however, the mechanism of DPBA as a probe is not well understood [25]. After 1 h of incubation at room temperature with shaking, the mycelia were removed from the well and visualized using fluorescence settings in an upright Eclipse Ni-E microscope (Nikon Metrology Inc., Brighton, MI, USA).

***Total antioxidant capacity (TAC) assay:*** AF3357 (1000 µL of 1 × 10^4^ CFU/mL initial concentration into 1% PDB medium pH 6.0 with 0.0001% Triton-X) with various flavonoid concentrations (0, 0.1, 0.01, 0.001, 0.0001, and 0.00001 µg/µL) and 1% DMSO (n = 4) was incubated for 24 h at 31 °C in the dark. Total antioxidant capacity was measured following the manufacturer’s instructions (total antioxidant capacity kit, Sigma-Aldrich, MAK187).

***Statistical analysis:*** Growth inhibition was determined from colony counts using Equation (1) below, as modified from Bekker et al. [26]. In this equation, a positive percentage of inhibition of growth indicates that the treated samples grew less than the control (DSMO only), while a negative value implies that the treated samples grew more than the control. Graphical creation of percentage of growth inhibition and data analysis were carried out using R (V4.1) [27]. A polynomial regression analysis was performed on the growth inhibition using R polynomial regression function. Any value above zero indicated inhibition of growth compared to the control, while below zero indicated the treatment grew more than the control.

Percentage of inhibition of proliferation = [average(control) − treatment]/average(control) × 100(1)

The aflatoxin B_1_ data were analyzed by performing one-way ANOVA of concentration of aflatoxin in relation to concentration of analyte, followed by a Tukey HSD test [28]. These were performed for each flavonoid separately. Sample quantiles and theoretical quantile graphs (Q-Q) graphs were generated for each analysis to determine normality in the data. MTT data were analyzed using the ratio of OD (560 nm)/OD (600 nm) regression formula that included adjusted R^2^ and *p*-value; log_2_ concentration was only used for scaling the figure, not for the regression analysis performed using R (V4.1) [27]. Total antioxidant capacity (TAC) was analyzed by generating a standard curve using the Trolox standards included in the kit and by determining the amount of antioxidant capacity in the sample in nmols of Trolox equivalents. For TAC analysis, the absorbance measurements were corrected by subtracting the absorbance measured from flavonoid treatment without fungi from the absorbance measured from fungi treated with the same flavonoid. One way ANOVA followed by a Tukey HSD test were used to determine differences in TAC using R (V4.1) [27]. 

## 3. Results

### 3.1. Proliferation Assays Show That Flavonoids Can Modulate Number of Fungal Colonies

**Figure 1:**
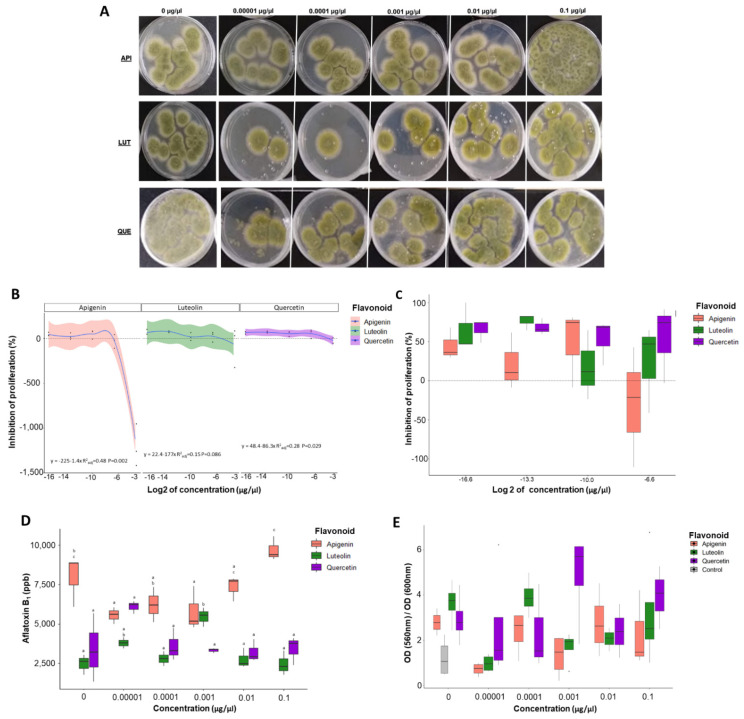
**Effect of flavonoids on proliferation, aflatoxin production, and cell metabolism of *A. flavus* 3357.** Biological assays performed with AF3357 strain exposed to apigenin, luteolin, and quercetin at 0 (1% DMSO only), 0.00001, 0.0001, 0.001, 0.01, and 0.1 µg/µL concentrations. (**A**) Pictures of Petri dishes from proliferation assays after 3 days of incubation. (**B**) Regression analysis of inhibition of proliferation and flavonoid concentration. Regression formula includes adjusted R^2^ and *p*-value; log_2_ concentration was only used for scaling, not for regression. (**C**) Box plots of inhibition of proliferation (highest concentration (0.1 µg/µL) was excluded). (**D**) Concentration of aflatoxin B1 (ppb). (**E**) MTT assay results expressed as the ratio of OD 560 nm/OD 600 nm. Key color legend represents chemical used; salmon: apigenin, green: luteolin, purple: quercetin, and gray: control (0% DMSO). Different letters over box plots represent significant difference according to Tukey HSD assay (*p* < 0.05) for aflatoxin B_1_ performed separately by flavonoid. Box plot whiskers depict the maximum and minimum without outliers, and the box depicts median and first and third quantile distribution. n = 3 per treatment. For Figure 1E, the dots are outliers.

**Figure 2:**
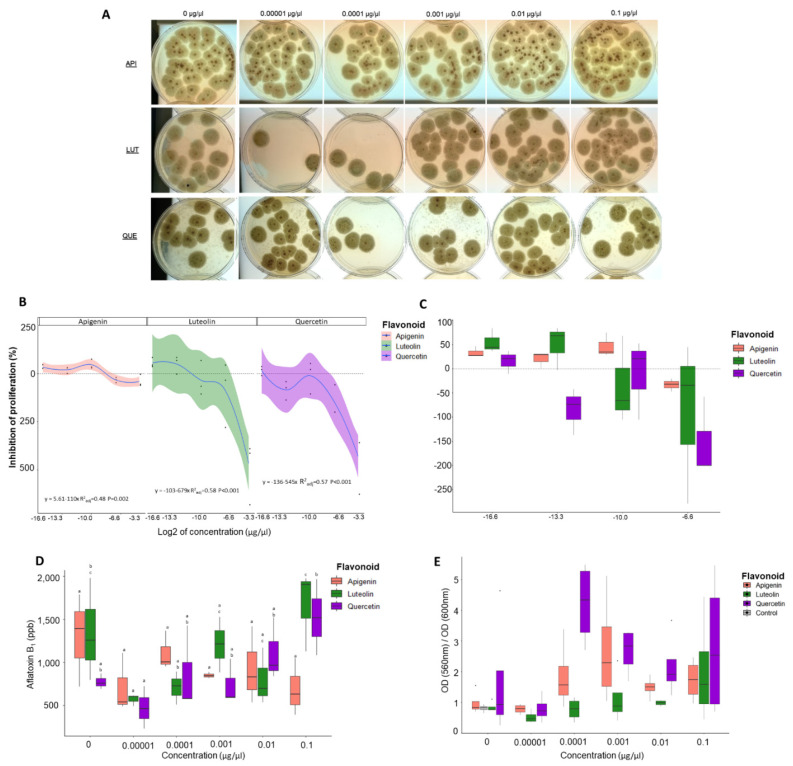
**Effect of flavonoids on proliferation, aflatoxin production, and cell metabolism of *A. flavus* 70-GFP.** Biological assays performed with AF70 (GFP tagged) strain exposed to apigenin, luteolin, and quercetin at 0 (1% DMSO only), 0.00001, 0.0001, 0.001, 0.01, and 0.1 µg/µL concentrations. (**A**) Pictures of Petri dishes from proliferation assays. (**B**) Regression analysis of inhibition of proliferation and flavonoid concentration. Regression formula includes adjusted R^2^ and *p*-value; log_2_ concentration was only used for scaling, not for regression. (**C**) Box plots of inhibition of proliferation (highest concentration (0.1 µg/µL) was excluded). (**D**) Concentration of aflatoxin B1 (ppb). (**E**) MTT assay results expressed as the ratio of OD 560 nm/OD 600 nm. Key color legend represents chemical used, with salmon: apigenin, green: luteolin, purple: quercetin, and gray: control (0% DMSO). Different letters over box plots represent significant difference according to Tukey HSD assay (*p* < 0.05) for aflatoxin B_1_ performed separately by flavonoid. Box plot whiskers depict the maximum and minimum without outliers, and the box depicts median and first and third quantile distribution. n = 3 per treatment. For Figure 2E, the dots are outliers.

Polynomial regression analysis showed significant correlation of inhibition of proliferation (see Equation (1)) when AF3357 was treated for 12 h with apigenin (R^2^_adj_ = 0.83, *p*-value < 0.001) and quercetin (R^2^_adj_ = 0.41, *p*-value = 0.021) (Figure 1). Similar results were seen for AF70 (apigenin R^2^_adj_ = 0.53, *p*-value = 0.002, luteolin R^2^_adj_ = 0.75, *p*-value < 0.001) and quercetin (R^2^_adj_ = 0.71, *p*-value < 0.001, Figure 2). These significant correlations mean that after 12 h of fungal exposure to quercetin and apigenin at 0.00001, 0.0001, and 0.001 µg/µL concentrations, there was inhibition of proliferation as a recovery response (Figure 1 and Figure 2). When AF3357 was treated for 12 h with 0.1 and/or 0.01 µg/µL of apigenin (−1210%; −29.8%), luteolin (−70%), and quercetin (−24%), we observed increased fungal proliferation (Figure 1). This promotion of proliferation was similar for AF70 when treated for 12 h with 0.1 and/or 0.01 µg/µL apigenin (−39%; 33.1%), luteolin (−494.7%; −89.5%), and quercetin (−447.4%; −152.6%) (Figure 2). In our regression analysis, concentration of flavonoid could significantly explain the inhibition of fungal proliferation. However, some regression models showed low R^2^_adj_, meaning there was high degree of variation in the model that cannot be explained by the flavonoid effects alone (Figure 1 and Figure 2). In summary, apigenin and quercetin showed inhibition of fungal proliferation at low concentrations with both AF3357 and AF70 fungal strains, while high concentrations of apigenin, luteolin, and quercetin (0.01 and 0.1 µg/µL) promoted fungal proliferation. Furthermore, despite not being significantly different, the dosage-dependent threshold showed different trends between fungal strains, namely, low levels of apigenin led to higher proliferation inhibition effect on AF3357 than AF70, while low levels of quercetin led to higher proliferation inhibition effect in AF70 than AF3357 (Figure 1 and Figure 2). 

### 3.2. Flavonoids Significantly Alter Aflatoxin Production in A. flavus

AF3357 treated for 12 h with low levels of apigenin (0.00001 µg/µL) led to significantly lower production of AFB_1_ (*p*-value = 0.0009; Figure 1 and Figure S1, Document S1) compared to the control. AF70 treated with apigenin also showed a trend to lower production of AFB_1_ (*p*-value = 0.07; Figure 2 and Figure S1, Document S1). AF70 treated for 12 h with luteolin (0.00001 µg/µL) led to significantly lower production of AFB_1_ compared to the control (*p*-value = 0.00826; Figure 2 and Figure S1, Document S1), while low levels of quercetin (0.00001 µg/µL) led to significantly lower production of AFB_1_ compared to the highest level of quercetin (0.1 µg/µL) (*p*-value = 0.02; Figure 2 and Figure S1, Document S1). Treating AF3357 with the highest levels of apigenin (0.1 µg/µL) led to a significant increase in production of AFB_1_ compared to the lowest treatment levels (*p*-value = 0.0029; Figure 1 and Figure S1, and Document S1). Similarly, AF70 treated with luteolin and quercetin at 0.1 µg/µL concentration led to higher AFB_1_ compared to treatments at low level (*p*-value = 0.029 and *p*-value = 0.04, respectively; Figure 2 and Figure S1). Overall, *A. flavus* exposed to low levels of apigenin and luteolin (0.00001 µg/µL) tended to decrease AFB_1_ production, while high levels of apigenin, luteolin, and quercetin (0.1 µg/µL) tended to increase AFB_1_ production. This effect by flavonoids could be due to these compounds acting as signaling molecules [17] and/or antioxidants [18,29].

### 3.3. Luteolin Significantly Affects the Ratio of Metabolic Rate to Fungal Growth Rate

We used an MTT assay to measure cellular metabolic activity as an indicator of cell viability. Viable cells contain NAD(P)H-dependent oxidoreductase enzymes that can reduce the MTT reagent (yellow) to formazan (purple) [30]. This reduction depends on the cellular metabolic activity due to NAD(P)H flux. In summary, if cells have low metabolic rates, reduction of MTT would be minimal. Because the flavonoids could affect cell growth, we avoided using higher OD_600_ readings in our calculations due to the presence of more cells instead of metabolically active cells; as such, we used the ratio of MTT OD to growth OD (560 nm/600 nm).

The ratio of metabolic rate to fungal growth showed that there were no significant differences between fungi treated with 1% DMSO and 0% DMSO (Figure 1 and Figure 2). At low concentrations of flavonoids, the metabolic rate/growth ratio tended to be lower compared to higher flavonoid concentration for both AF3357 and AF70 (Figure 1, Figure 2 and Figure S1). There were variations in these trends at different concentration of flavonoids, which could be due to feedback loops arising from fungal consumption and excretion of bioactive molecules and their catabolites from the media [31,32]. Further investigation is required to understand this variability and dosage-dependent responses. Overall, there was a trend of increased metabolic rate/growth ratio as the flavonoid treatment increased in concentration (Figure 1, Figure 2 and Figure S1). The *p*-values of the regression analysis showed that only AF70 treated with luteolin significantly affected the ratio of metabolic rate to fungal growth (*p*-value = 0.028, Figure S1).

### 3.4. Flavonoids Affect the Physical Properties of the Fungal Cell Wall

**Figure 3:**
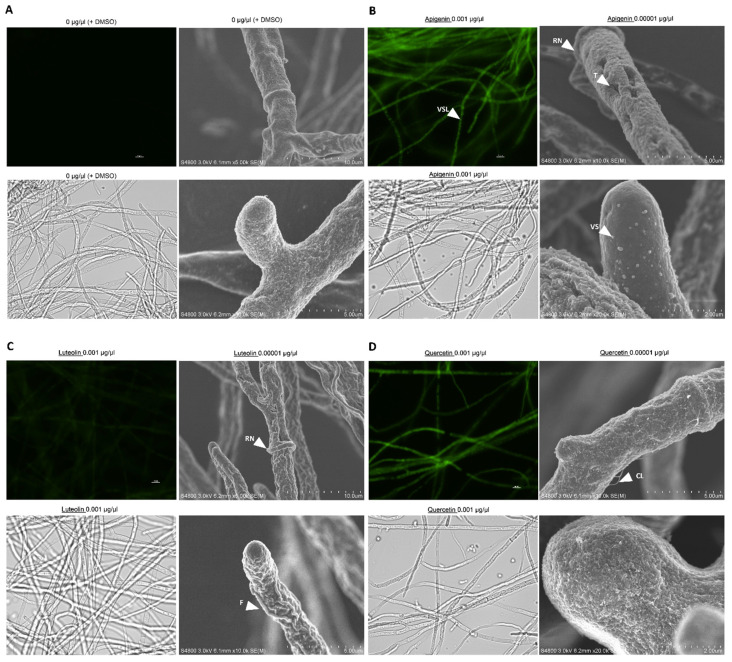
**Flavonoids accumulate in spherical structures inside mycelia and disrupt physical properties of the cell wall.** DPBA assay (visualized with fluorescence), light microscopy, and SEM of AF3357 strain exposed to flavonoids. (**A**) Control (0 µg/µL with 1% DMSO); (**B**) 0.001 and 0.00001 µg/µL apigenin; (**C**) 0.001 and 0.00001 µg/µL luteolin; and (**D**) 0.001 and 0.00001 µg/µL quercetin. White bars and ticks represent scale of the microscope image. In the figures, indicate RN: ring-like structure, VS: vesicle structure, VSL: vesicle-like structure, F: fold, CL: cloud-like peels, and T: tear-like structure.

Bright-field and fluorescence microscopy analyses were unable to detect any physical differences in the mycelia treated with flavonoids (Figure S2). The resolution of the bright-field and fluorescence microscopy images at lower magnifications was too low to determine the presence of cell damage in the mycelium (Figure S3). Hence, evidence of damage was further investigated at higher magnifications with scanning electron microscopy (SEM). We investigated AF3357 with SEM to visualize the effects of flavonoid treatments on hyphal cell surface and cell wall properties at high resolution (2–50 µm). Apigenin and luteolin treatments (0.01 and 0.00001 µg/µL, respectively) showed significant physical alterations of the fungal hyphae, such as shrinkage, invaginations, and tears, compared to the control (Figure 3 and Figure S4). In addition, these two flavonoids led to accumulation of vesicles-like structures on the cell surface, apical cells, and formation of a ring around the region where the septum would be localized (Figure 3 and Figure S4). Interestingly, quercetin treatments showed a different type of physical damage, with the surface of the cell wall appearing to peel off in strings and cloud-like structures (Figure 3 and Figure S4). Quercetin treatments lacked vesicle-like structures with similar diameter as the ones observed in apigenin and luteolin treatments (Figure 3 and Figure S4). Due to the differences of flavonoid effects on the surface of fungal hyphae, it is possible that different flavonoids have different modes of action.

### 3.5. Flavonoids Are Localized in Vesicle-like Structures on Hyphae

We used AF3357 and DPBA staining to visualize flavones and flavonols inside or on the surface of the mycelium. Apigenin, luteolin, and quercetin were observed on the surface of the hyphae (Figure S5). High flavonoid concentrations (0.001 to 0.1 µg/µL) showed fluorescent spherical structures inside the hyphae; these spheres could be vesicle-like structures (Figure 3 and Figure S4). 

### 3.6. Low Concentrations of Flavonoids Decrease Antioxidant Capacity

**Figure 4:**
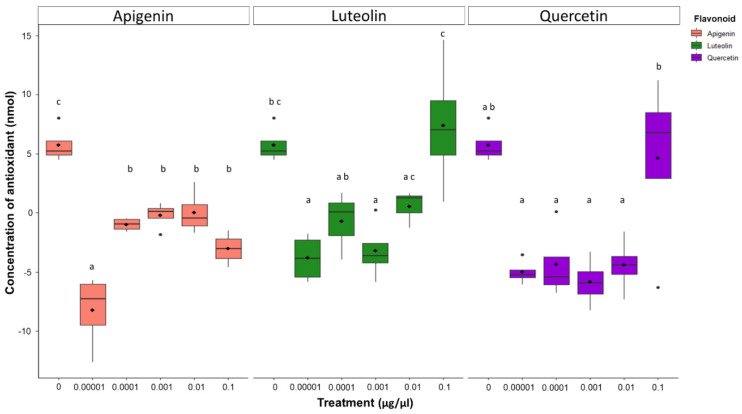
**Flavonoids change total antioxidant capacity in the environment.** Total antioxidant capacity (TAC) assay performed with AF3357 strain exposed to apigenin, luteolin, and quercetin at 0 (1% DMSO only), 0.00001, 0.0001, 0.001, 0.01, and 0.1 µg/µL concentrations. Letters above box plots indicate the Tukey HSD test results with *p* < 0.05 performed for each flavonoid. Box plot whiskers depict the maximum and minimum without outliers, and the box depicts median and first and third quantile distribution. n = 4 per treatment. Color legend represents chemical used, with salmon: apigenin, green: luteolin, and purple: quercetin. Concentration of antioxidant (nmol) in sample is given using the standard curve for Trolox.

Total antioxidant capacity (TAC) analysis showed that AF3357 strain treated with low levels of apigenin and luteolin (0.00001 µg/µL) had lower antioxidant capacity (−8.2 and −3.8 nmol, respectively) compared to the control (5.7 nmol) and the highest concentration (0.1 µg/µL) of flavonoids (−3.1 and 7.4 nmol, respectively) (Figure 4). Low TAC could indicate a decrease in antioxidants in the environment, which could translate into higher levels of reactive oxygen species (ROS) and reactive nitrogen species (RNS) that can cause oxidative damage to the fungus and/or alter signaling pathway cascades. Our results indicate that apigenin and luteolin treatments can change the TAC by either decreasing it in low concentration treatments compared to the control or increasing it in high concentration treatments compared to lower concentrations (Figure 4). This modulation of TAC agrees with the idea that flavonoids themselves can behave as scavengers of ROS [18]. Moreover, the fact that we saw lower TAC in lower concentrations compared to the control could indicate that, in addition to scavenging ROS, these molecules could be altering REDOX state by other mechanisms. 

## 4. Discussion

We previously established that corn lines resistant to aflatoxin contamination produce more flavonoids under *A. flavus* infection in kernels [6]. As such, we aimed to investigate the effect that apigenin, luteolin, and quercetin have on *A. flavus* NRRL 3357. We found that treating the fungus with a high concentration of flavonoids increased its proliferation measured as a recovery response to flavonoid exposure, while low concentrations inhibited fungal proliferation in a dosage-dependent manner that was also fungal strain specific (Figure 1 and Figure 2). These results partially agree with previously published data that demonstrated the antimicrobial potential of flavonoids in *Fusarium oxysporum* f. sp. *lycopersici* [10]. To further characterize the effect of flavonoids on *A. flavus,* we looked at fungal cell surface morphological responses using SEM and found that all flavonoids tested led to specific alterations to cell wall integrity, such as shrinkage, invaginations, tears, peeling, and vesicle formation. These results agree with previous studies that utilized transmission electron microscope (TEM) images of *A. parasiticus* treated with flavonoids and showed mycelial damage, such as multiple lipid vesicle formation stimulated by naringenin; degradation or rupture of plasmalemma, cell wall deformation, and vesicle formation by neohesperidin; and agglutination of fibrillar layer, formation of dense grains in the inner wall, disruption of nuclear membranes, and formation of vesicles by quercetin [8]. Furthermore, shrinkage of the cell wall along the hyphae and around the septum has been reported for SEM in *F. graminearum* treated with thymol [33] and cell-free supernatants of *Pediococcus pentosaceus* [34]. Similar shrinkage or cracking of the cell wall observed with SEM were reported in *A. fumigatus* treated with wogonin, one of the major flavones in Ou-gon (*Scutellaria* root extract) extracts [35]. It is possible that the molecular mechanisms linked to the physical damage caused by flavonoids vary in relation to the chemical and the fungal species. Further research is required to investigate the relationship of flavonoids to fungal cell wall damage and mode of action.

We found that two flavonoids (apigenin and luteolin) were localized in vesicle-like structures (Figure 3 and Figure S5). Production of vesicles by *A. parasiticus* treated with naringenin has been observed previously [8]. Vesicle-like structures were detected on the surface of the fungal hyphae in our SEM examination (Figure 3). We hypothesize that the vesicles could contain heterostructures composed of macromolecules, such as proteins, that could bind to flavonoids. We also hypothesize that due to flavonoid effects on fungal proliferation, REDOX state, and their aggregation into vesicle-like structures, flavonoids may be functioning as signaling molecules as suggested by others [17]. 

To further characterize the effects of flavonoids on aflatoxin production by *A. flavus,* published evidence showed inhibition of growth [13] and AFB_1_ production following treatment of *A. flavus* with anthocyanidins and flavonoids [12]. We observed similar inhibition of AFB_1_ production by flavonoids at the lowest concentration (0.00001 µg/µL), while we observed increased AFB_1_ compared to the DMSO control in the highest concentration (0.1 µg/µL). In our studies, both flavonoid treatments and controls had the same concentration of DMSO (1% = 140 mM). In-vitro-grown *F. culmorum* and *F. graminearum* metabolized flavonoids to their derivatives and decreased mycotoxin accumulation [9]. Our results suggest that the modulation in AFB_1_ production is a result of flavonoid treatment, and the mechanisms of this modulation could be linked to oxidoreduction processes.

The relationship and interplay between flavonoids, aflatoxins, and oxidoreduction in *A. flavus* appears complicated. Flavonoids that have ROS-scavenging ability are needed to control development in eukaryotic cells [19]. *A. flavus* treated with ROS showed elevated expression of genes encoding enzymes involved in phenylpropanoid metabolic pathways that can modulate aflatoxin production in vitro [29]. Aflatoxins have been hypothesized to be scavengers of ROS in *A. parasiticus*, which can be linked to enhanced resilience to harsh environments that could play a role in fungal adaptation [36]. We determined that *A. flavus* treated with a low concentration (0.00001 µg/µL) of apigenin, luteolin, and quercetin had lower antioxidant capacity compared to the control (Figure 4), and interestingly, these flavonoid concentrations led to inhibition of fungal proliferation and inhibition of aflatoxin production (Figure 1 and Figure 2). Modulation of oxidoreduction levels supports the observation that flavonoids themselves can behave as scavengers of ROS [18]. However, some of the flavonoid concentrations at which we observed changes in antioxidant capacity (e.g., 0.00001 µg/µL) are likely too low for the flavonoids to be effective ROS quenchers [19], indicating that flavonoids could be altering REDOX state by other mechanisms, such as affecting signaling pathways linked to growth and toxin production. It is noteworthy that under high concentration treatments of flavonoids, we saw an increase in aflatoxin concentration and fungal proliferation. Thus, it is possible that aflatoxin and flavonoids had a synergistic effect to increase the total antioxidant capacity and allow the fungus to have better growth and development. 

Flavonoids act as signaling molecules capable of modulating microbial growth [37]. Corn plants have been shown to modulate rhizosphere-growth-promoting microbiota through the flavone composition of their root exudates [37]. In *Triadica sebifera*, differences in root exudate flavonoids can enhance arbuscular mycorrhizal (AM) fungal associations [38]. Two flavanones, hesperetin and naringenin, were found to be the most effective plant-to-bacteria signal molecules in the interaction between *Rhizobium leguminosarum*, pea (*Pisum sativum* L.) and lentil (*Lens culinaris* L.) by inducing *nod* gene expression and preactivation of *nod* genes [39]. Our results support the idea that phenylpropanoid derivatives, such as flavonoids, can modulate oxidoreduction reactions, fungal proliferation, and production of aflatoxin in *A. flavus*. We hypothesized that flavonoids could act as potential signaling molecules that are taken inside the fungal cells in vesicle-like structures, thus changing the oxidative state of the environment, leading to changes in proliferation, development, and aflatoxin production and altering the fungal cell wall integrity. Due to the location of the vesicle-like structures, we hypothesized that flavonoids might be aggregating and forming heterostructures with other biomolecules that are taken into the cells possibly involved in signaling pathways. Further research of exosomes and endosomes produced by the fungus in response to low and high concentrations of flavonoids will help illuminate important molecular pathways. Inclusion of other biochemical and molecular features, such as feedback loops due to fungal consumption of molecules in the media [31,32], could lead to better understanding of the variability and dosage-dependent responses of fungi to flavonoids. Some questions remain: What are the molecular mechanisms and signaling pathways involving flavonoids that control aflatoxin production and fungal proliferation during REDOX changes? How do these changes affect the physical integrity of the cell wall? How do REDOX changes regulate those signaling pathways? Further research could illuminate the complete molecular mechanisms of flavonoids and their effects on cell wall integrity, oxidoreduction levels, fungal proliferation, and aflatoxin production in *A. flavus*. 

## Data Availability

Data supporting reported results can be found in Supplementary Materials.

## Supplementary Materials

The following supporting information can be downloaded at: https://www.mdpi.com/article/10.3390/jof8111211/s1, **Figure S1.** Tukey HSD results for aflatoxin B_1_ concentration from AF3357 treated with (A) apigenin, (B) luteolin, and (C) quercetin and from AF70 (GFP tagged) treated with (D) apigenin, (E) luteolin, and (F) quercetin. Regression analysis of cell metabolism (MTT) from (G) AF3357 and (H) AF70 treated with flavonoids at 0 (1% DMSO only), 0.00001, 0.0001, 0.001, 0.01, and 0.1 µg/µL concentrations. MTT assay was measured as OD 560 nm/OD 600 nm. Letters above box plots indicate the Tukey HSD test results with *p* < 0.05. Error bars represent standard errors. Regression formula includes adjusted R^2^ and *p*-value; log_2_ concentration was only used for scaling, not for regression. Key color legend represents chemical used, with salmon: apigenin, green: luteolin, and purple: quercetin. **Figure S2. Flavonoids do not affect the microscopic attributes from the fungi.** Fluorescence microscopy images taken from AF70 strain exposed to flavonoids at 0 (1% DMSO only), 0.00001, 0.0001, 0.001, 0.01, and 0.1 µg/µL concentrations. (A) Apigenin, (B) luteolin, and (C) quercetin. White bar represents scale of the magnification. **Figure S3. Mycelia treated with flavonoids.** Fluorescence microscopy images taken from AF70 strain exposed to flavonoids at 0 (1% DMSO only), 0.00001, 0.0001, 0.001, 0.01, and 0.1 µg/µL concentrations. (A) Apigenin, (B) luteolin, and (C) quercetin. White bar represents scale of the magnification. **Figure S4. Flavonoids disrupt fungal cell wall integrity.** SEM of AF3357 strain exposed for 24 h to flavonoids. (A) Control (0 µg/µL with 1% DMSO); (B) 0.01 and 0.00001 µg/µL apigenin; (C) 0.01 and 0.00001 µg/µL luteolin; and (D) 0.01 and 0.00001 µg/µL quercetin. White bar (2–50 µm) and ticks represent scale of the magnification. In the figures, white and black arrows indicate RN: ring-like structure; VS: vesicle structure; F: fold; CL: cloud-like peels; T: tear-like structure. **Figure S5. Flavonoids localize outside and inside fungal mycelia.** DPBA assay visualized with fluorescence microscopy from AF3357 strain exposed to flavonoids at 0, 0.00001, 0.0001, 0.001, 0.01, and 0.1 µg/µL concentrations. (A) Controls (left two images: 0 µg/µL with 1% DMSO, right two images: 0 µg/µL without 1% DMSO), (B) apigenin, (C) luteolin, and (D) quercetin. White bar represents scale of the magnification; bar = 10 µm. In figures, white arrows indicate VSL: vesicle-like structure. **Document S1.** Statistical analysis results of aflatoxin B_1_ accumulation from AF3357 and AF70 strains exposed to flavonoids and pictures of experimental set-up.
