# Supplementary material for: Flavonoids Modulate Aspergillus flavus Proliferation and Aflatoxin Production
Source: J Fungi (Basel). 2022 Nov 16;8(11):1211. doi: 10.3390/jof8111211 (PMC9693025; doi:10.3390/jof8111211)
Supplement: Supplementary file 1 [file jof-08-01211-s001.zip › jof-1960284-supplementary.pdf]

Journal of Fungi
MDPI

Supplemental Materials

Document S1:

Table: Molar concentrations of flavonoids.

Concentration (µg/µl or g/L)	Apigenin in µM (FW: 270g/mol)	Luteolin in µM (FW: 286.24g/mol)	Quercetin in µM (FW: 302.23g/mol)
0.00001	0.04	0.03	0.03
0.0001	0.37	0.35	0.33
0.001	3.70	3.49	3.31
0.01	37.04	34.94	33.09
0.1	370.37	349.36	330.87

Statistical analysis 1: Model and statistical analysis of aflatoxin B1 concentrations for *A. flavus* 3357 strain treated with apigenin:

	Df	Sum Sq	Mean Sq	F value	Pr(>F)
Concentration	5	41182652	8236530	9.676	0.000958 ***
Residuals	11	9363607	851237		

Signif. codes: p < 0.001 '***'; p > 0.001 '**'; p > 0.01 '*'; p > 0.05 '.'; p > 1 ' '

Pairwise comparisons using t tests with pooled SD

	0	1e-05	1e-04	0.001	0.01
1e-05	0.0275	-	-	-	-
1e-04	0.0915	1.0000	-	-	-
0.001	0.0406	1.0000	1.0000	-	-
0.01	0.5718	0.2967	0.8246	0.4452	-
0.1	1.0000	0.0029	0.0099	0.0043	0.0915

P value adjustment method: holm

Statistical analysis 2: Model and statistical analysis of aflatoxin B1 concentrations for *A. flavus* 3357 strain treated with luteolin:

	Df	Sum Sq	Mean Sq	F value	Pr(>F)
Concentration	5	20395391	4079078	10.65	0.000635 ***
Residuals	11	4214210	383110		

Signif. codes: p < 0.001 '***'; p > 0.001 '**'; p > 0.01 '*'; p > 0.05 '.'; p > 1 ' '

1 observation deleted due to missingness

Pairwise comparisons using t tests with pooled SD

	0	1e-05	1e-04	0.001	0.01
1e-05	0.3627	-	-	-	-
1e-04	1.0000	0.7194	-	-	-

0.001	0.0014	0.1475	0.0031	-	-
0.01	1.0000	0.7013	1.0000	0.0029	-
0.1	1.0000	0.3627	1.0000	0.0014	1.0000

P value adjustment method: holm

Statistical analysis 3: Model and statistical analysis of aflatoxin B1 concentrations for *A. flavus* 3357 strain treated with quercetin:

	Df	Sum Sq	Mean Sq	F value	Pr(>F)
Concentration	5	18558140	3711628	3.052	0.0526 .
Residuals	12	14595632	1216303		

Signif. codes: p < 0.001 '***'; p > 0.001 '**'; p > 0.01 '*'; p > 0.05 '.'; p > 1 ' '

Pairwise comparisons using t tests with pooled SD

	0	1e-05	1e-04	0.001	0.01
1e-05	0.15	-	-	-	-
1e-04	1.00	0.18	-	-	-
0.001	1.00	0.13	1.00	-	-
0.01	1.00	0.12	1.00	1.00	-
0.1	1.00	0.15	1.00	1.00	1.00

P value adjustment method: holm

Statistical analysis 4: Model and statistical analysis of aflatoxin B1 concentrations for *A. flavus* 70 (GFP tagged) strain treated with apigenin:

	Df	Sum Sq	Mean Sq	F value	Pr(>F)
Concentration	5	1295743	259149	2.721	0.0774 .
Residuals	11	1047764	95251		

Signif. codes: p < 0.001 '***'; p > 0.001 '**'; p > 0.01 '*'; p > 0.05 '.'; p > 1 ' '

Pairwise comparisons using t tests with pooled SD

	0	1e-05	1e-04	0.001	0.01
1e-05	0.14	-	-	-	-
1e-04	1.00	1.00	-	-	-
0.001	0.31	1.00	1.00	-	-
0.01	0.46	1.00	1.00	1.00	-
0.1	0.13	1.00	1.00	1.00	1.00

P value adjustment method: holm

Statistical analysis 5: Model and statistical analysis of aflatoxin B1 concentrations for *A. flavus* 70 (GFP tagged) strain treated with luteolin:

	Df	Sum Sq	Mean Sq	F value	Pr(>F)
Concentration	5	3092860	618572	5.607	0.00826 **
Residuals	11	1213495	110318		

Signif. codes: p < 0.001 '***'; p > 0.001 '**'; p > 0.01 '*'; p > 0.05 '.'; p > 1 ' '

Pairwise comparisons using t tests with pooled SD

	0	1e-05	1e-04	0.001	0.01
1e-05	0.070	-	-	-	-
1e-04	0.133	1.000	-	-	-
0.001	1.000	0.347	0.745	-	-
0.01	0.207	1.000	1.000	0.967	-
0.1	1.000	0.029	0.065	0.819	0.101

P value adjustment method: holm

Statistical analysis 6: Model and statistical analysis of aflatoxin B1 concentrations for *A. flavus* 70 (GFP tagged) strain treated with quercetin:

	Df	Sum Sq	Mean Sq	F value	Pr(>F)
Concentration	5	1966857	393371	3.368	0.0394 *
Residuals	12	1401585	116799		

Signif. codes: p < 0.001 '***'; p > 0.001 '**'; p > 0.01 '*'; p > 0.05 '.'; p > 1 ' '

Pairwise comparisons using t tests with pooled SD

	0	1e-05	1e-04	0.001	0.01
1e-05	1.00	-	-	-	-
1e-04	1.00	1.00	-	-	-
0.001	1.00	1.00	1.00	-	-
0.01	1.00	0.45	1.00	1.00	-
0.1	0.26	0.04	0.42	0.23	1.00

P value adjustment method: holm

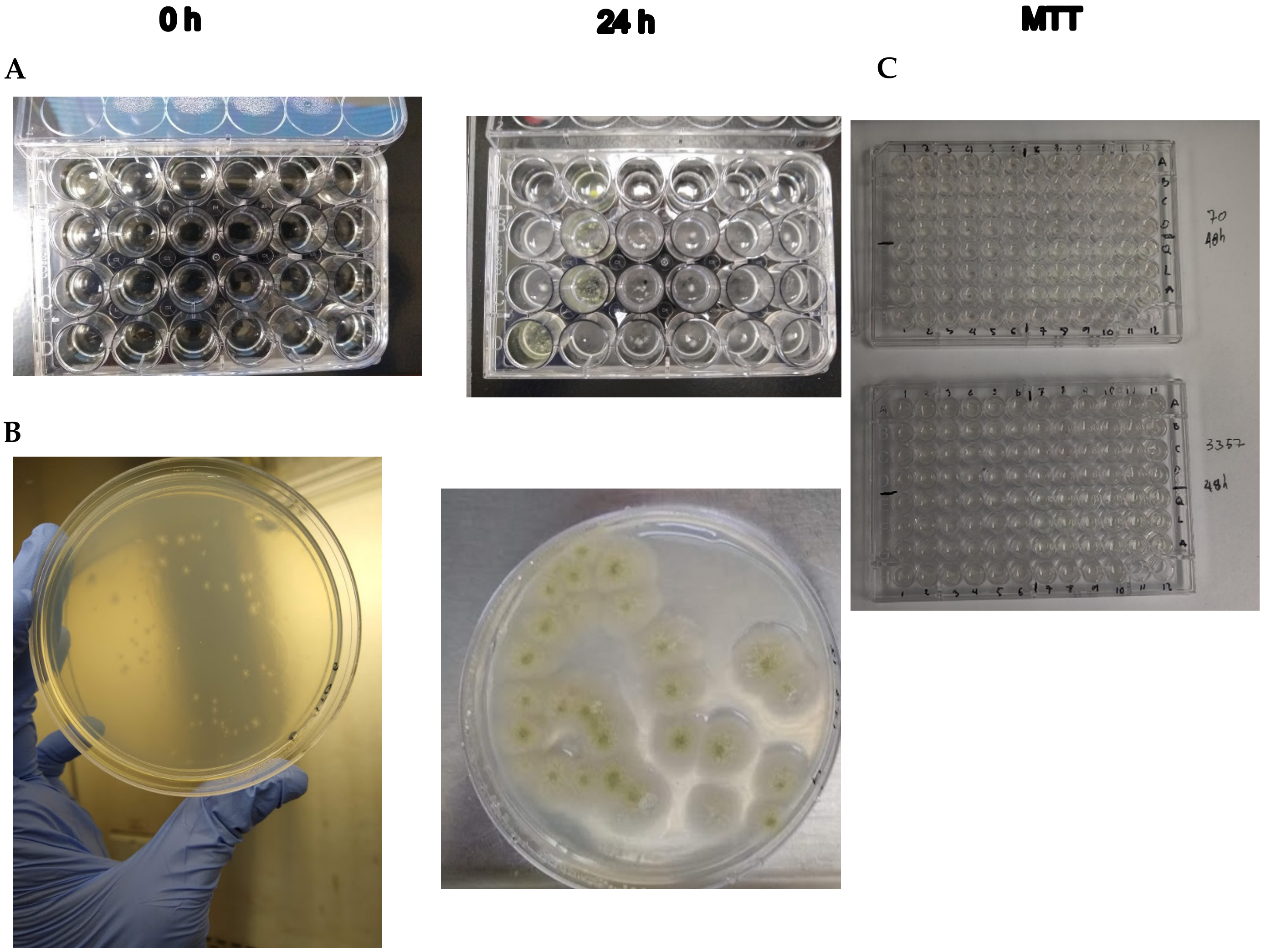

Pictures of experimental set up for (A) bioassay proliferation after 18 h of incubation at 31ºC in the dark, (B) counting of colonies from the proliferation assay and (C) MTT assays.

A

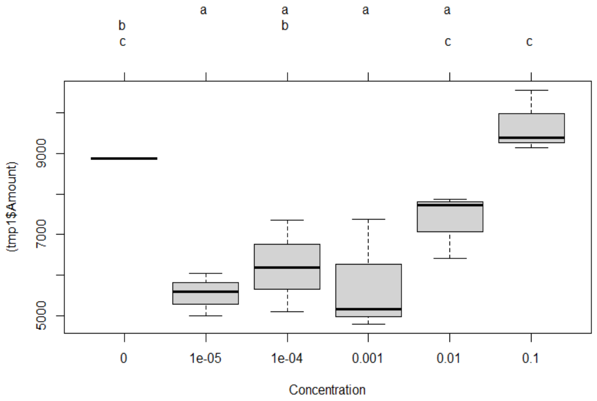
(tmp1$Amount)
Concentration

A′

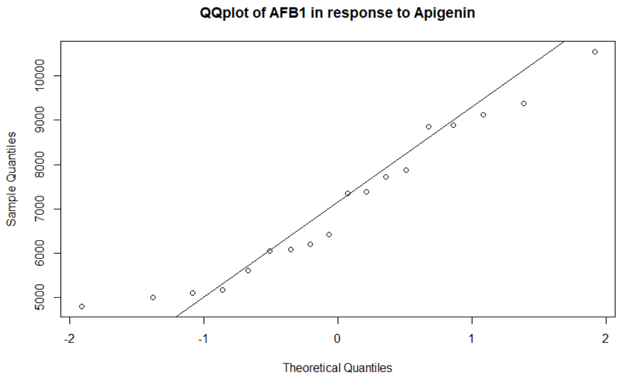
QQplot of AFB1 in response to Apigenin
Sample Quantiles
Theoretical Quantiles

B

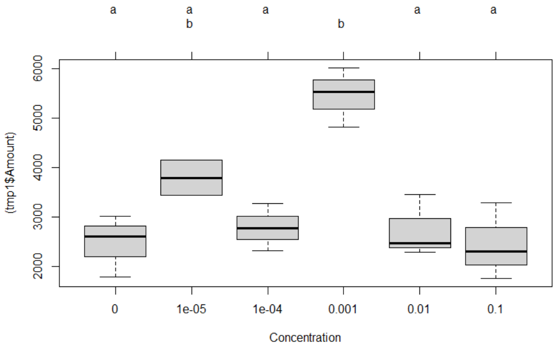
(tmp1$Amount)
Concentration

B′

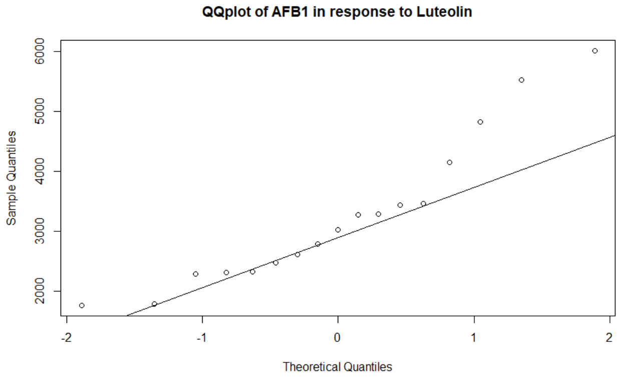
QQplot of AFB1 in response to Luteolin
Sample Quantiles
Theoretical Quantiles

C

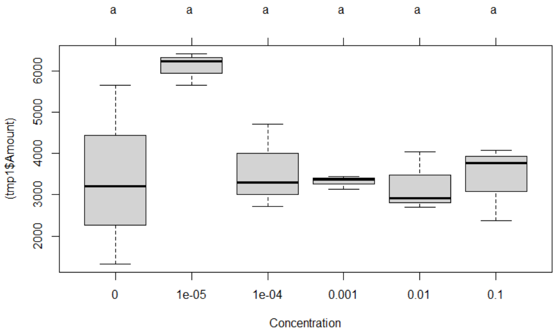
(tmp1$Amount)
Concentration

C′

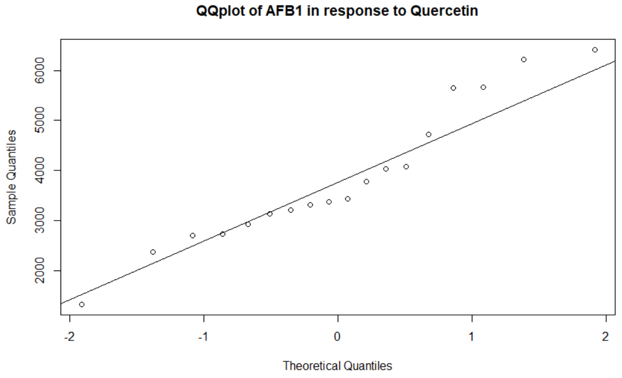
QQplot of AFB1 in response to Quercetin
Sample Quantiles
Theoretical Quantiles

D

D′

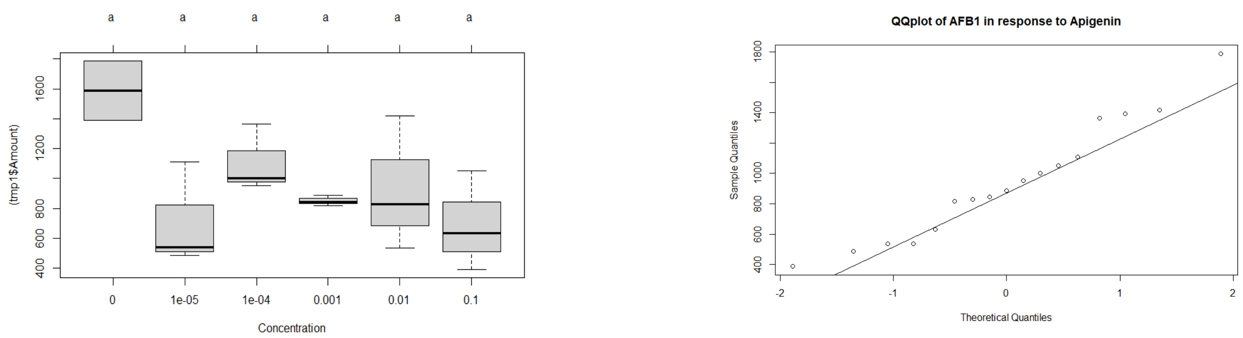
QQplot of AFB1 in response to Apigenin
(tmp1$Amount)
Concentration
Sample Quantiles
Theoretical Quantiles

E E′

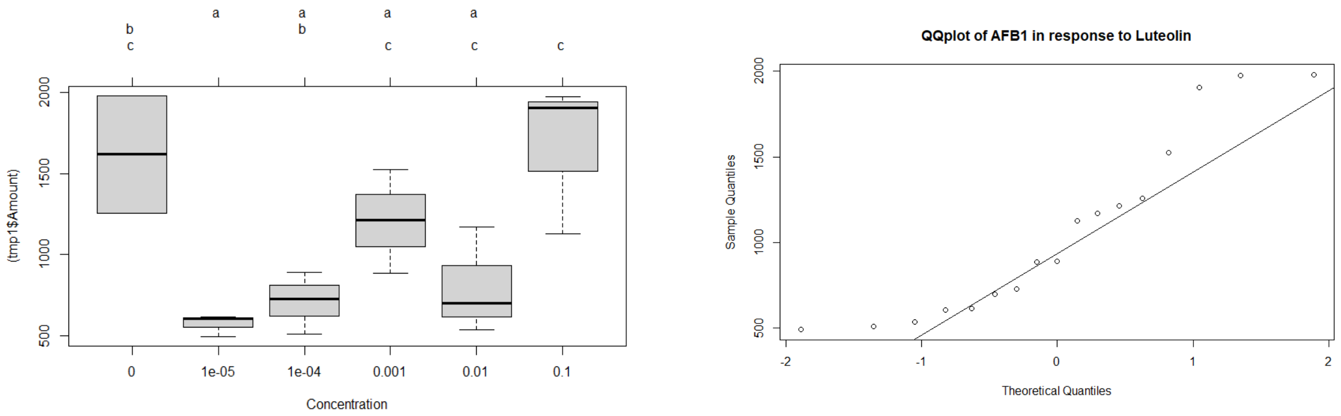
QQplot of AFB1 in response to Luteolin
(tmp1$Amount)
Concentration
Sample Quantiles
Theoretical Quantiles

F F′

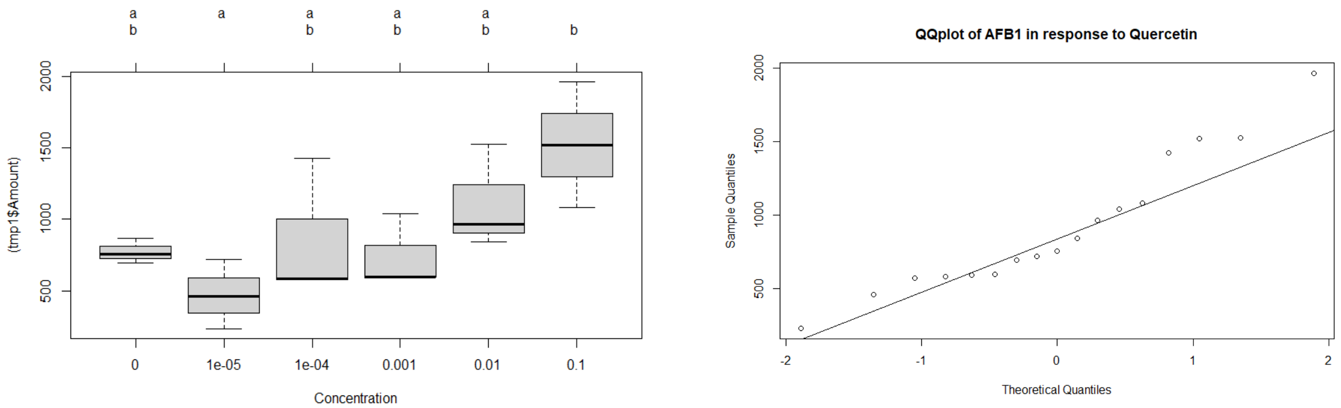
QQplot of AFB1 in response to Quercetin
(tmp1$Amount)
Concentration
Sample Quantiles
Theoretical Quantiles

G H

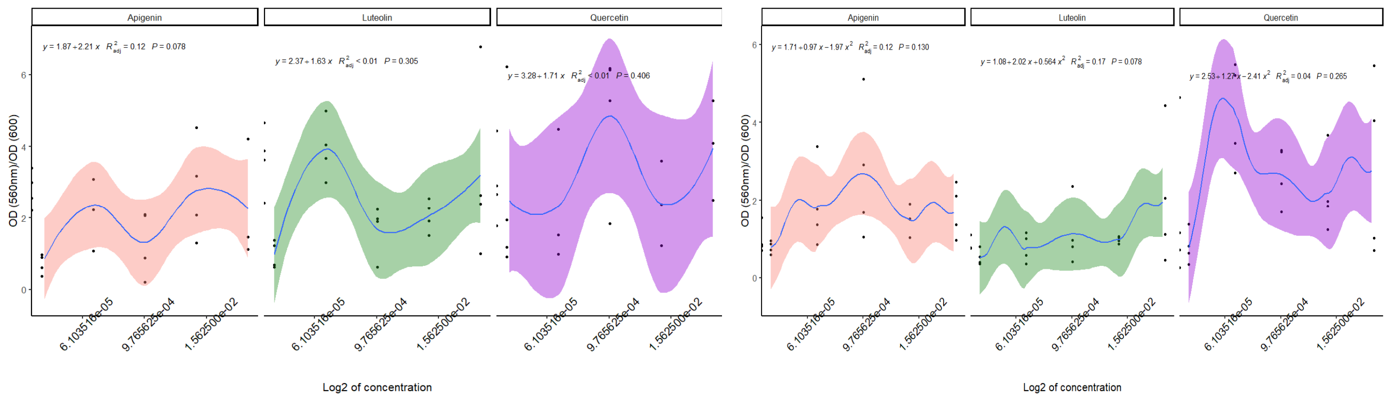
Apigenin
Luteolin
Quercetin
OD (560nm)/OD (600)
Log2 of concentration

Figure S1. Tukey HSD results for aflatoxin B_1 concentration from AF3357 treated with (A and A′) apigenin, (B and B′) luteolin, and (C and C′) quercetin and from AF70 (GFP tagged) treated with (D and D′) apigenin, (E and E′) luteolin, and (F and F′) quercetin. Regression analysis of cell metabolism (MTT) from (G) AF3357 and (H) AF70 treated with flavonoids at 0 (1% DMSO only), 0.00001, 0.0001, 0.001, 0.01, and 0.1 µg/µL concentrations. MTT assay was measured as OD 560 nm/OD 600 nm. Letters above box plots indicate the Tukey HSD test results with $p < 0.05$. Error bars represent standard errors. Regression formula includes adjusted R^2 and p-value; $\log_2$ concentration was only used for scaling, not for regression. Key color legend represents chemical used, with salmon: apigenin, green: luteolin, and purple: quercetin.

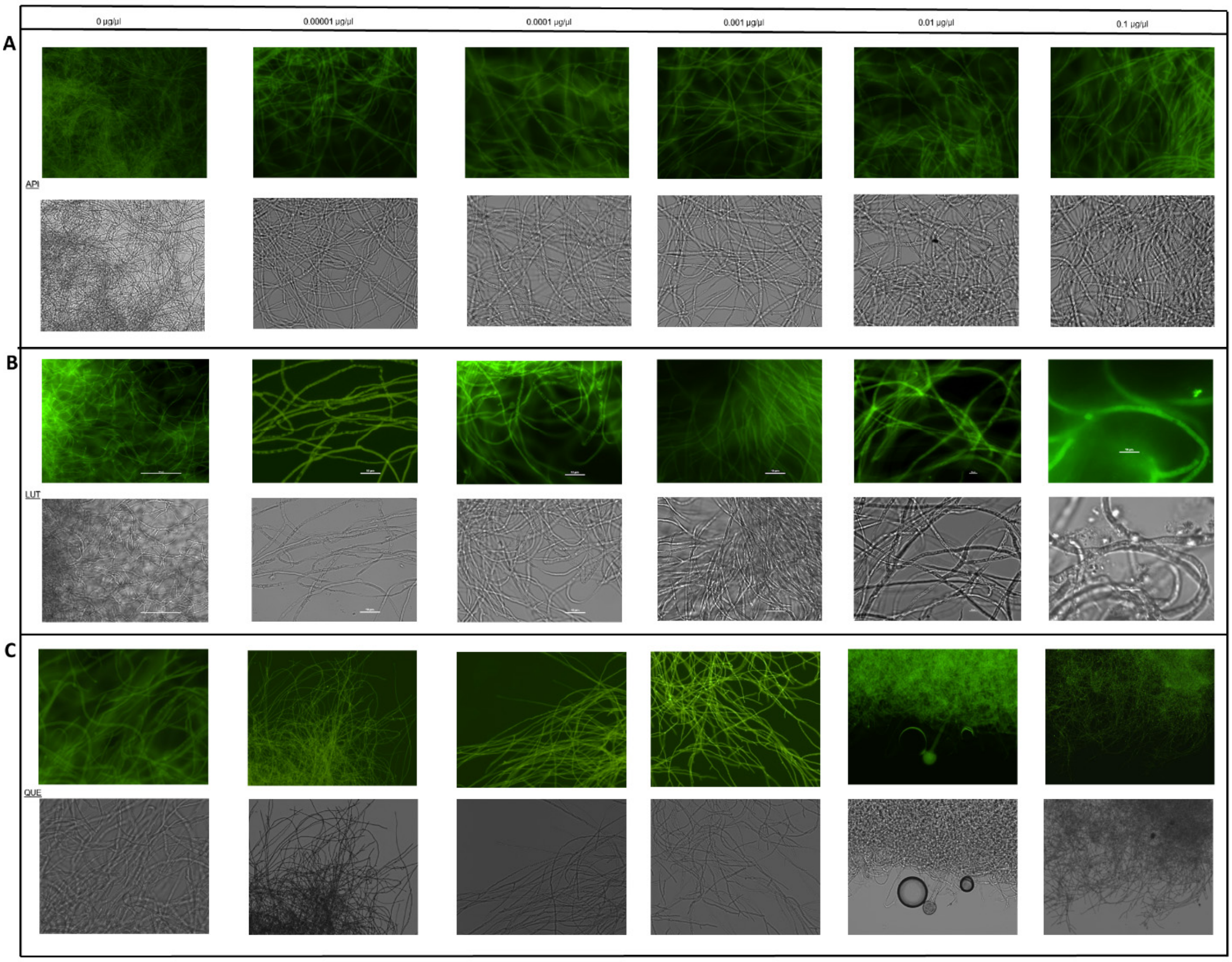

Figure S2. Flavonoids do not affect the microscopic attributes from the fungi. Fluorescence microscopy images taken from AF70 strain exposed to flavonoids at 0 (1% DMSO only), 0.00001, 0.0001, 0.001, 0.01, and 0.1 µg/µL concentrations. (A) Apigenin, (B) luteolin, and (C) quercetin. White bar represents scale of the magnification. The scale bars (from left to right from top to bottom):

100um; 50um; 50um; 50um; 50um; 100um;

100um; 50um; 50um; 50um; 50um; 100um;

100um; 10um; 10um; 10um; 10um; 10um;

100um; 10um; 10um; 10um; 10um; 10um;

50um; 100um; 100um; 100um; 100um; 100um;

50um; 100um; 100um; 100um; 100um; 100um;

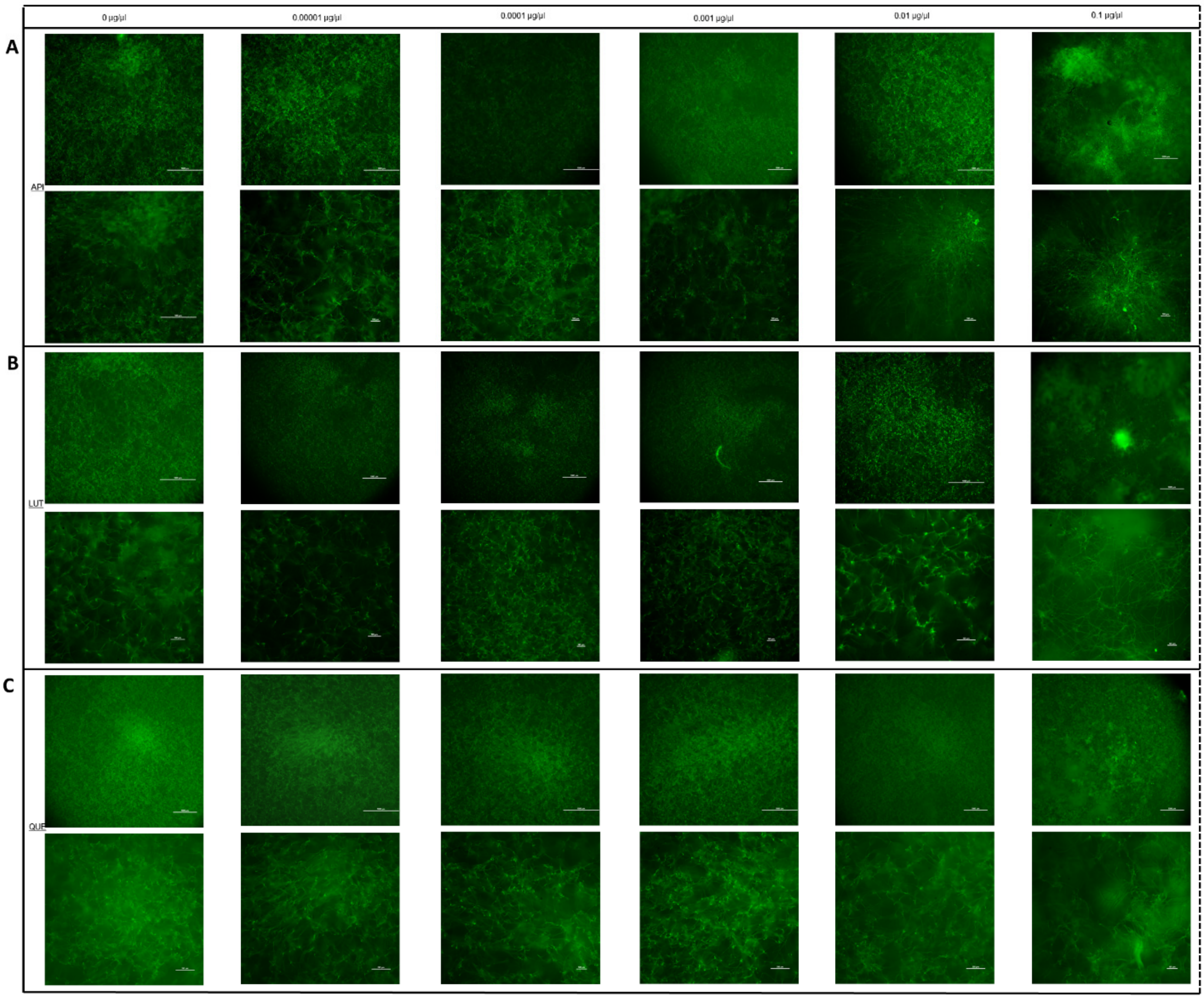

Figure S3. Mycelia treated with flavonoids. Fluorescence microscopy images taken from AF70 strain exposed to flavonoids at 0 (1% DMSO only), 0.00001, 0.0001, 0.001, 0.01, and 0.1 µg/µL concentrations. (A) Apigenin, (B) luteolin, and (C) quercetin. White bar represents scale of the magnification. The scale bars (from left to right from top to bottom):
1000um; 1000um; 1000um; 1000um; 1000um; 1000um;
500um; 100um; 100um; 100um; 100um; 100um;
1000um; 1000um; 1000um; 1000um; 1000um; 1000um;
100um; 100um; 100um; 100um; 100um; 100um;
1000um; 1000um; 1000um; 1000um; 1000um; 1000um;
200um; 200um; 100um; 200um; 200um; 100um;

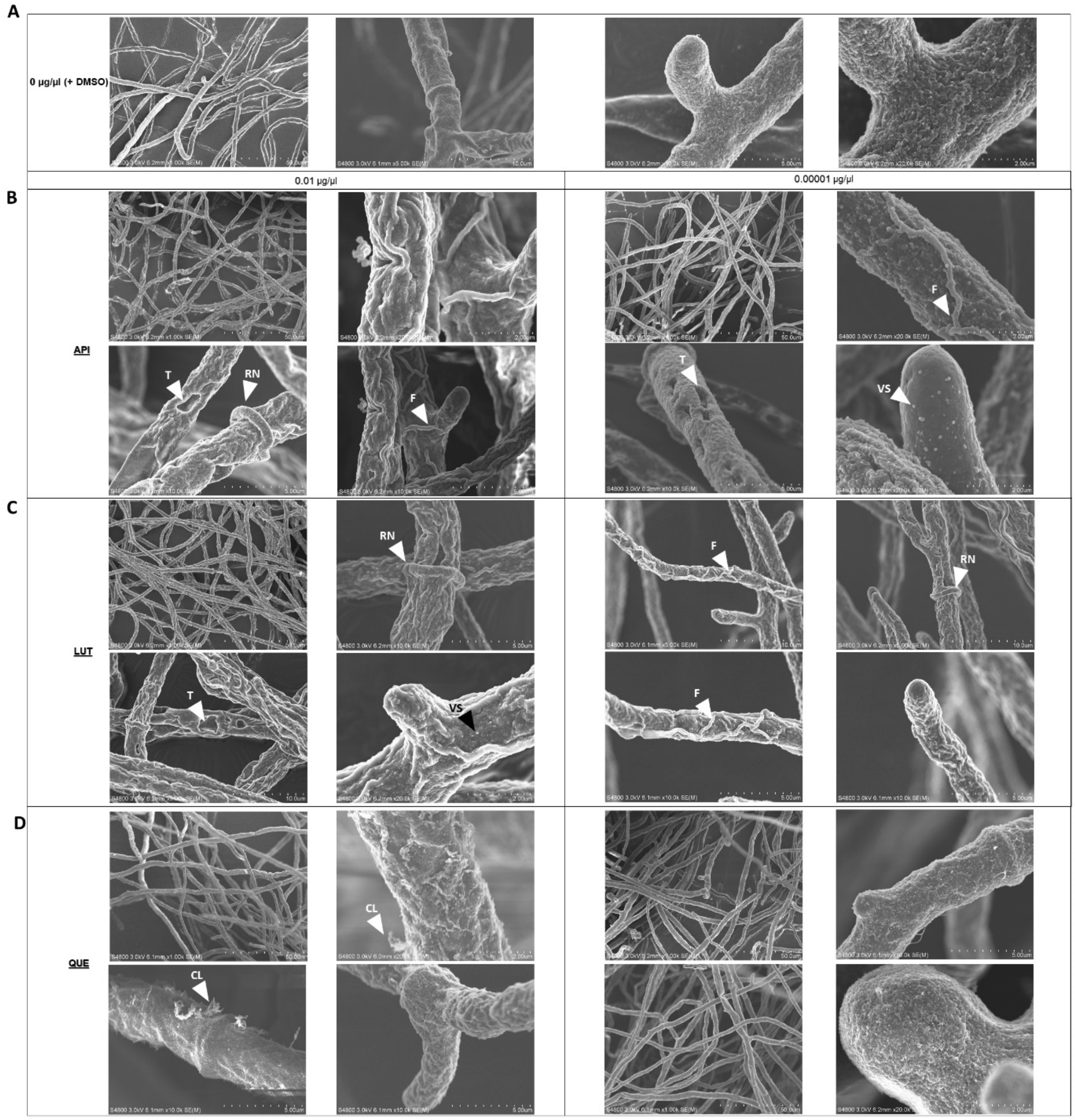

Figure S4. Flavonoids disrupt fungal cell wall integrity. SEM of AF3357 strain exposed for 24 h to flavonoids. (A) Control (0 µg/µL with 1% DMSO); (B) 0.01 and 0.00001 µg/µL apigenin; (C) 0.01 and 0.00001 µg/µL luteolin; and (D) 0.01 and 0.00001 µg/µL quercetin. White bar (2–50 µm) and ticks represent scale of the magnification. In the figures, white and black arrows indicate RN: ring-like structure; VS: vesicle structure; F: fold; CL: cloud-like peels; T: tear-like structure. The scale bars (from left to right from top to bottom):

50um; 10um; 5um; 2um;

50um; 2um; 50um; 2um;

5um; 5um; 5um; 2um;

50um; 5um; 10um; 10um;

10um; 2um; 5um; 5um;

50um; 2um; 50um; 5um;

5um; 5um; 50um; 2um;

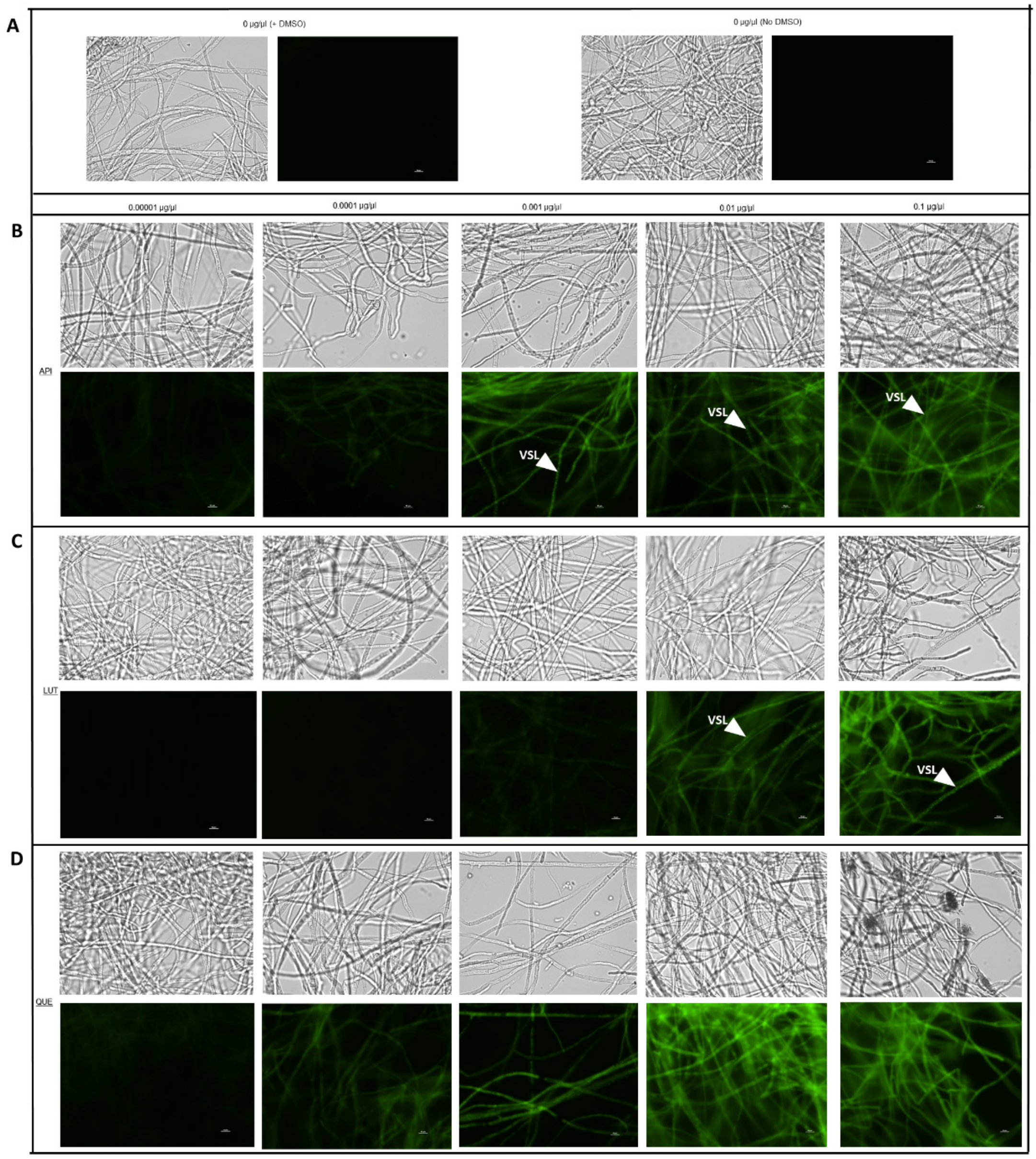

Figure S5. Flavonoids localize outside and inside fungal mycelia. DPBA assay visualized with fluorescence microscopy from AF3357 strain exposed to flavonoids at 0, 0.00001, 0.0001, 0.001, 0.01, and 0.1 μg/μL concentrations. (**A**) Controls (left two images: 0 μg/μL with 1% DMSO, right two images: 0 μg/μL without 1% DMSO), (**B**) apigenin, (**C**) luteolin, and (**D**) quercetin. White bar represents scale of the magnification; bar = 10 μm. In figures, white arrows indicate VSL: vesicle-like structure. The scale bars is 10 um.
